# Cultural Scripts of Trauma in East Africa Predicted by Cultural and Intrapersonal Factors: Path Analysis of a New Psychopathological Concept

**DOI:** 10.1002/cpp.70225

**Published:** 2026-01-23

**Authors:** Yucong Wen, Andreas Maercker, Nathanael Adank, Celestin Mutuyimana

**Affiliations:** ^1^ Division of Psychopathology and Clinical Intervention, Department of Psychology University of Zurich Zürich Switzerland

**Keywords:** cultural scripts, East Africa, general self‐efficacy, self‐mastery, social axioms, trauma

## Abstract

Cultural factors play a central role in shaping responses to traumatic events. However, most trauma research has been conducted in Western populations, and culturally specific trauma sequelae remain underexplored in other contexts. This study applied the framework of cultural scripts of trauma (CSTs) to examine how cultural values, intrapersonal factors and symptoms of posttraumatic stress disorder (PTSD) and disturbances in self‐organization (DSO) are interrelated in an East African context. This cross‐sectional study used convenience sampling. Data from East African trauma survivors were analysed using structural equation modelling and path analysis to investigate links among social axioms, general self‐efficacy, self‐mastery, CSTs, and PTSD and DSO symptoms. Social axioms (social complexity, reward for application and religiosity) predicted growth‐related CSTs more strongly than self‐efficacy or self‐mastery. CSTs such as frame of mind and body‐related symptoms were positively associated with PTSD severity. Mediation analyses indicated that CSTs partially explained the association between social axioms and PTSD symptoms. Notably, CSTs such as family stress and cognitive disruption were not linked to PTSD or DSO, suggesting culturally specific trauma responses not captured by current diagnostic criteria. Findings provide empirical support for the CSTs framework in an East African setting. The study highlights the importance of integrating cultural values and culturally specific symptom expressions into trauma assessment and intervention, and advances the development of culturally sensitive models of adaptation to trauma by identifying both unique and cross‐cultural dimensions of trauma responses.

## Summary

- Cultural scripts of trauma (CSTs) offer a framework for understanding how trauma is expressed in culturally specific ways, extending beyond Western diagnostic categories.
- In East African survivors, cultural values (e.g., religiosity, social complexity, reward for application) strongly shaped trauma responses, highlighting the importance of integrating cultural worldviews into assessment and treatment.
- Certain CSTs (e.g., ‘frame of mind’, ‘body‐related symptoms’) were linked to PTSD severity, suggesting that clinicians should pay attention to culturally shaped symptom expressions that may otherwise be overlooked.
- Some trauma responses (e.g., ‘family stress’, ‘cognitive disruption’) were not tied to standard PTSD or DSO symptoms, underlining the need for culturally sensitive diagnostic tools and interventions.

## 1 Introduction

Conceptual frameworks (e.g., Hwang et al. 2008; Jobson and O'Kearney 2009; Wong et al. 2010) and empirical studies (e.g., Chang et al. 2017) underscore the importance of culture—shared patterns of beliefs, values and behaviours that define and differentiate groups (Hofstede 1998; Kluckhohn and Strodtbeck 1961) —for mental health. Cultural factors are increasingly recognized as critical for understanding posttraumatic stress disorder (PTSD) and complex posttraumatic stress disorder (CPTSD) (Heim et al. 2022).

Evidence from diverse regions indicates that culture shapes the perception and expression of posttraumatic sequelae (Maercker and Augsburger 2019), the manifestation of trauma‐related disorders (Agbayani‐Siewert et al. 1999; Heim et al. 2022; Knefel et al. 2019; Perilla et al. 2002) and PTSD prevalence (Dückers and Brewin 2018). Today, both the International Classification of Diseases (ICD‐11, World Health Organization 2019) and the Diagnostic and Statistical Manual of Mental Disorders (DSM‐5‐TR, American Psychiatric Association 2022) acknowledge culture in influencing how individuals interpret, express and respond to trauma, via general cultural considerations or inclusion of cultural syndromes and idioms of distress.

It has been argued that cultural concepts of distress can represent the posttraumatic reality of trauma survivors better than the established concepts of PTSD and CPTSD (e.g., Hinton et al. 2019). Sociocultural information can increase diagnostic suitability and provide a better tool for understanding human distress and suffering (Alarcón et al. 2009). Thus, investigating cultural psychological factors beyond standard diagnostic criteria of PTSD and CPTSD can yield a more nuanced understanding of traumatic experiences. Although PTSD and CPTSD are designed as pan‐cultural diagnostic constructs, they may serve more effectively as intermediate frameworks to guide the development of culturally sensitive, region‐specific formulations (Heim et al. 2022).

Most research on posttraumatic sequelae has been conducted in Western contexts (Kirmayer and Ryder 2016), leading to the underrepresentation of culturally specific trauma symptoms in standardized PTSD and CPTSD assessments (Owczarek et al. 2020; Vallières et al. 2018). Existing definitions may omit significant trauma manifestations that do not align with Western diagnostic frameworks (Maercker and Heim 2019; Nwoye 2015; Pole et al. 2008). Cross‐cultural evidence further indicates that findings from Western settings may not generalize to non‐Western populations (Kirmayer 2012; Whitley et al. 2011). Consequently, PTSD and CPTSD diagnoses and treatments, grounded in Western symptom sets, have been criticized for limited cultural adaptability (Hinton and Lewis‐Fernandez 2011; Lewis‐Fernandez and Kirmayer 2019), leaving culturally specific posttraumatic sequelae in non‐Western populations insufficiently understood (Maercker and Heim 2019).

To address this gap, the concept of cultural scripts of trauma (CSTs, Chentsova‐Dutton and Maercker 2019) has emerged as a valuable framework. CSTs describe dynamic, culturally shaped responses to trauma, offering a context‐sensitive perspective that enhances understanding of trauma experiences and informs culturally tailored interventions (Chentsova‐Dutton and Ryder 2020; Napier et al. 2014). Specifically, CSTs encompass culturally specific posttraumatic symptoms and signs, typically expressed as states of suffering and recovery, often sequentially or causally interconnected (Chentsova‐Dutton and Maercker 2019; Napier et al. 2014). Studies on CSTs have identified CSTs elements in distinct cultural contexts; for instance, Bachem et al. (2024) in German‐speaking Switzerland and Mutuyimana and Maercker (2023) in East Africa. These findings capture dominant signs of suffering and the complexity of trauma sequelae beyond those listed in ICD or DSM criteria.

Building on an international series of qualitative substudies (Bachem et al. 2024; Maercker et al. 2024; Mutuyimana and Maercker 2023; Papava et al. 2025; Yu et al. 2026), a recent study in four East African countries produced a content‐validated set of the following five ‘signs and symptoms’ clusters: cognitive symptoms (e.g., negative self‐perceptions, insecurity and emotional fragility), frame of mind (a more negative view of the world following traumatic experiences), body‐related symptoms (e.g., sleep disturbances, headaches and tingling sensations), growth (e.g., increased self‐determination, gratitude and kindness) and family stress (e.g., challenges in managing family resources and heightened negative perceptions of the family) (Mutuyimana and Maercker 2026). This data set provides the basis for examining how cultural and intrapersonal factors predict these clusters.

Social axioms represent generalized cultural beliefs about the self, social and physical environment and the spiritual world, shaped by and embedded within cultural contexts (Bond, Leung, Au, Tong, de Carrasquel, et al. 2004). Five pan‐cultural axioms have been identified: social cynicism, reward for application, social complexity, religiosity and fate control (Bond, Leung, Au, Tong, de Carrasquel, et al. 2004; Bond, Leung, Au, Tong, and Chemonges‐Nielson 2004; Leung et al. 2002). As a worldview and value‐based orientation, social axioms have been linked to COVID‐19–related psychopathology (Murashcenkova 2023) and are thought to influence the development of cultural syndromes and CSTs (Maercker and Heim 2019).

In contrast to cultural variables, intrapersonal factors include general self‐efficacy (GSE) and self‐mastery. GSE reflects one's belief in the ability to achieve desired outcomes through personal action (Bandura 1982) and has been inversely associated with posttraumatic stress symptoms (e.g., Gallagher et al. 2020; Luszczynska et al. 2009). By facilitating adaptive coping, GSE can reduce posttraumatic stress levels (Benight and Bandura 2004). Similarly, self‐mastery—a psychological resource reflecting confidence in managing life and confronting stressors—has been shown to buffer negative effects and improve posttraumatic outcomes (Bebanic et al. 2017; Benight and Bandura 2004; Bowlin and Baer 2012; Gilbar et al. 2010; Hall et al. 2010; Moore et al. 2015; Pearlin and Schooler 1978; Pooley et al. 2013; Zanbar et al. 2021).

Populations in East Africa have experienced cumulative, repetitive and collective traumas in recent decades, including the 1994 Rwandan genocide, increasing risk for psychopathology. Trauma‐related disorders, such as PTSD and CPTSD, have been documented in these populations (Bapolisi et al. 2020; Mugisha et al. 2015; Mutuyimana et al. 2023), with CPTSD extending PTSD symptoms to include disturbances in self‐organization (affective dysregulation, negative self‐concept and relational disturbances).

This study examined relationships among CSTs, cultural factors (social axioms), intrapersonal factors (GSE, self‐mastery) and PTSD/CPTSD symptoms in four East African countries—Rwanda, Kenya, Tanzania and Uganda—that share histories of traumatic exposure and similar cultural contexts (Rasmussen et al. 2014). Specifically, this study investigated (1) the relationships between CSTs ‘signs and symptoms’ clusters in East Africa (i.e., cognitive symptoms, frame of mind, body‐related symptoms, growth and family stress; Mutuyimana and Maercker 2026); (2) how social axioms, GSE and self‐mastery predict CSTs; (3) the predictions of CSTs and PTSD or CPTSD (as construct validation replication of a previous study by Mutuyimana and Maercker 2026).

This study proposes two hypotheses and two exploratory questions. Hypothesis 1: social axioms predict CSTs more strongly than GSE and self‐mastery. Hypothesis 2: CSTs are positively associated with PTSD and CPTSD symptom severity. Exploratory Question 1: What are the relationships between social axioms and CSTs, PTSD and CPTSD symptom severity? Exploratory Question 2: Do CSTs mediate the associations between social axioms, GSE, self‐mastery and PTSD/CPTSD symptom severity?

## 2 Method

### 2.1 Study Design and Sample

This cross‐sectional study employed convenience sampling, recruiting participants via online platforms (Instagram, WhatsApp) and field data collection in Rwanda, Kenya, Tanzania and Uganda, with support from research assistants. Eligibility criteria included experiencing traumatic events, citizenship of the respective country and proficiency in the questionnaire language; individuals currently undergoing traumatic events or mental health crises were excluded. Sample size was determined via power analysis for SEM (Schumacker and Lomax 2022). For the proposed model with 14 latent and 58 observed variables, a minimum of 815 participants was required to achieve α = 0.05 and power = 0.80.

The final sample comprised 1208 participants: Rwanda (*n* = 320), Kenya (*n* = 220), Tanzania (*n* = 412) and Uganda (*n* = 256) (Mutuyimana and Maercker 2026). Questionnaires were administered in Kinyarwanda (Rwanda), Swahili (Tanzania) and participants' choice of Swahili or English (Uganda); in Kenya and Uganda, English was the most accessible language. The online survey required approximately 30 min. Only participants providing electronic informed consent were included. Ethical approval was obtained from the University of Rwanda, College of Medicine and Health Sciences (Approval No. 396/CMHS IRB/2022) and Daystar University, Kenya (Approval No. DU‐ISERC/09/05/2023/00086).

### 2.2 Measures

#### 2.2.1 CSTs

CSTs in East Africa were assessed by the 15‐item Cultural Scripts of Trauma Inventory (CSTI), which was developed after a thorough mixed‐method process (Mutuyimana and Maercker 2026). The CSTI was divided into the following five subscales: cognitive symptoms, frame of mind, body‐related symptoms, growth and family stress. Each subscale consisted of three items.

Participants rated items on a 5‐point Likert scale (0 = *strongly disagree* to 4 = *strongly agree*), with higher scores indicating more pronounced culturally related posttraumatic symptoms. The CSTI has demonstrated good reliability and validity (Mutuyimana and Maercker 2025). In this study, Cronbach's α was 0.86 for the total scale and ranged from 0.67 to 0.78 for the subscales.

#### 2.2.2 PTSD and CPTSD Symptoms

These symptoms were measured using the International Trauma Questionnaire (ITQ; Cloitre et al. 2018). It examined PTSD symptoms according to ICD‐11 with two items each on re‐experiencing, avoidance and sense of threat. It also examined disturbances in self‐organization (DSO) symptoms with two items each on affect dysregulation, negative self‐concept and disturbances in relationships and several items on functional impairment. ITQ used a 5‐point Likert scale ranging from 0 (*not at all*) to 4 (*extremely*). The total score ranged from 0 to 72; PTSD and DSO subscale scores ranged from 0 to 36. Respondents were instructed to answer PTSD and DSO items based on their past‐month symptoms. In this study, the internal consistency estimates of ITQ were excellent. Cronbach's α was 0.87 for ITQ, 0.83 for PTSD and 0.86 for DSO.

#### 2.2.3 Social Axioms

The Social Axioms Survey (SAS; Leung et al. 2002) measured beliefs about people and the world by 15 items. It is commonly regarded as a cultural orientations measure. The SAS consists of five subscales: social cynicism, social complexity, reward for application, religiosity, and fate control. For each subscale, we selected three items with the highest factor loading according to Leung et al. (2012, 840–841). Items of SAS scoring used a 5‐point scale, ranging from 1 (*strongly disagree*) to 5 (*strongly agree*). The total score ranged from 15 to 75. Because of low Cronbach's α in our sample for social cynicism (0.58) subscales, we only used one item (*People who become rich and successful forget the people who helped them along the way*) and for fate control with α = 0.43 two items (*Fate determines a person's success in life; The people whom a person will love in his or her life are determined by fate*), resulting in these Cronbach's α: 0.74 for reward for application, 0.78 for social complexity, 0.64 for fate control and 0.81 for religiosity.

#### 2.2.4 GSE

We used the GSE subscale of the Self‐Efficacy Scale (Sherer et al. 1982). We used nine items, which had the highest factor loading on the GSE dimension to measure participants' ability to cope with the daily life stressors (1 = *strongly disagree* to 5 = *strongly agree*). Cronbach's α = 0.78 in this study.

#### 2.2.5 Self‐Mastery

The seven‐item Self‐Mastery Scale (Pearlin and Schooler 1978) assessed perceived control over life circumstances. After deleting two items with poor performance (*I can do just about anything I really set my mind to*; *What happens to me in the future mostly depends on me*), Cronbach's α = 0.61.

#### 2.2.6 Demographic Characteristics

Demographic data were also collected, including year of birth, gender, country, marital status, education level, occupation, monthly income, religion and location.

### 2.3 Statistical Analyses

**FIGURE 1:**
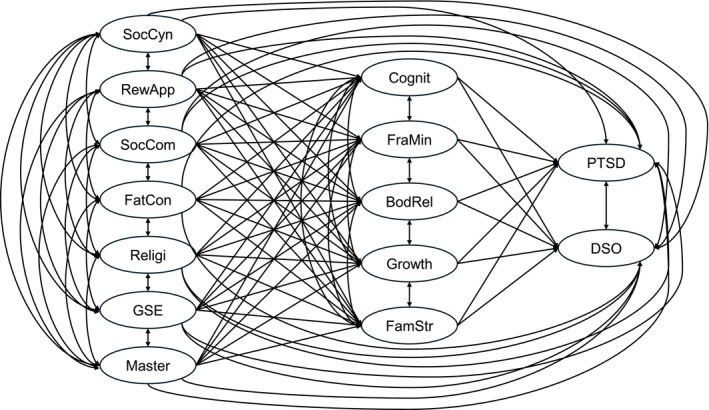
Hypothesized initial structural equation modelling diagram. BodRel = body‐related symptoms, Cognit = cognitive symptoms, DSO = disturbances in self‐organization, FamStr = family stress, FatCon = fate control, FraMin = frame of mind, GSE = general self‐efficacy, Master = self‐mastery, PTSD = posttraumatic stress disorder, Religi = religiosity, RewApp = reward for application, SocCom = social complexity, SocCyn = social cynicism.

We tested the proposed conceptualization model addressing the relationships between (a) social axioms, GSE, self‐mastery and (b) CSTs on explaining posttraumatic outcomes (PTSD and DSO symptoms). There was no missing data or outlier found in this study, so all the 1208 records were analysed. Statistical analyses in this study proceeded in five steps. First, descriptive statistics of all variables were computed. Second, we compared the PTSD and DSO symptoms severity, as well as symptom groups of PTSD and DSO among four countries by using one‐way analysis of variance (ANOVA) as a control analysis to estimate equivalence across the four countries. Third, Pearson's correlation was used to examine correlations among the studied variables. Fourth, we conducted a series of hierarchical multiple regressions and relative importance analyses using the Lindeman, Merenda and Gold method (LMG) for five CSTI subfactors to examine whether SAS subfactors explained more variance in CSTs, PTSD and DSO symptoms severity than intrapersonal variables (GSE and self‐mastery). Fifth, we performed SEM analysis. Two separate theoretical models were tested to examine the relationships between (a) social axioms, GSE, self‐mastery, (b) CSTs and (c) PTSD and DSO symptoms. We firstly tested the core model (see Figure 1), which included five social axioms subfactors, self‐mastery and GSE in the first layer (exogenous variables). The second layer includes the following five CSTI subfactors as mediators: cognitive symptoms, frame of mind, body‐related symptoms, growth and family stress. In the third layer, PTSD and DSO were included as outcome variables.

All exogenous variables were modelled to predict all mediators and outcome variables; all mediators in the second layer were modelled to predict outcome variables. Secondly, we also tested an alternative model in comparison to the core model. The alternative model was the same as the core model except that it regarded CSTI—the mediators—as only two variables in the second layer (the first variable was created by summing up the negative aspects of CSTI—cognitive symptoms, frame of mind, body‐related symptoms and family stress; the second variable was the growth subfactor of CSTI). SEM was estimated using maximum likelihood to test the hypothesized relationships in the two models mentioned above.

For all path coefficients, standardized estimates (and the 95% CI of the estimates) were computed. Statistical significance was determined at *p* < 0.05. The model fit was evaluated by using these goodness‐of‐fit indices: χ^2^/df within acceptable range of 1–5. The comparative fit index (CFI), Tucker‐Lewis Index (TLI) and the root mean square error of approximation (RMSEA; Kline 2016) were also used. CFI and TLI of at least 0.90 and an RMSEA below 0.08 suggested a good fit to the data (Hu and Bentler 1999; Kline 2016). To supplement the RMSEA, we also presented its 90% CI (i.e., an estimate of its precision); a CI with an upper bound below 0.08 indicated a good model fit (MacCallum et al. 1996). Standardized root mean square residual (SRMR) below 0.08 was indicative of a good fit (Hu and Bentler 1999). Finally, we examined the Akaike and Bayesian information criterion (AIC and BIC, respectively) indices; lower values indicate better model fit. We considered *p* values smaller than 0.05 to be significant. For the better model, we used the bootstrapping technique recommended by Preacher and Hayes (Preacher and Hayes 2008) to estimate the path coefficients and standard errors of direct and indirect effects using the maximum likelihood estimator. The estimations were calculated based on bootstrapping for 1000 replications.

We calculated Cronbach's α for each scale and compared symptoms severity between four countries by using SPSS 26 (IBM Corp. 2019). Other analyses were performed by R Version 4.4.2 (R Core Team 2024) and R Studio (Posit Team 2024).

## 3 Results

### 3.1 Descriptive Statistics and Bivariate Correlations

Table 1 presents the socio‐demographic characteristics of the sample. The majority of participants were female (63.2%), with childhood trauma (35.7%, *n* = 431) and genocide (19.6%, *n* = 237) being the most commonly reported traumatic experiences.

**TABLE 1:** Demographic characteristics.

Variable	*n*/years	%/SD
Age (years)	32.64	10.85
Gender		
Male	436	36.1
Female	763	63.2
Not identified	9	0.7
Country		
Rwanda	320	26.5
Uganda	256	21.2
Tanzania	412	34.1
Kenya	220	18.2
Marital status		
Single	628	52.0
Married	451	37.3
Separated/divorced	129	10.7
Education level		
Primary school	104	8.6
Secondary school	234	19.4
Bachelor's degree	723	59.9
Master's and PhD degree	147	12.2
Occupation		
Student	375	31.0
Unemployed	170	14.1
Self‐employed/employed	663	54.9
Monthly income		
< 100$	527	43.6
$100–300$	300	24.8
$300–500$	206	17.1
500–$1000$	111	9.2
> 1000$	64	5.3
Religion		
Non religion	28	2.3
Christian	962	79.6
Muslim	204	16.9
Traditional religion	14	1.2
Traumatic exposure		
Genocide	237	19.6
War	141	11.7
Exile	16	1.3
Childhood trauma	431	35.7
Imprisonment	115	9.5
Terrorism	81	6.7
Rape	187	15.5
Location		
Rural	330	27.3
Urban	878	72.7

Group comparisons indicated that participants from Tanzania exhibited the highest severity of PTSD, DSO and related symptom clusters (see Table A1 in the Appendix). However, as all effect sizes comparing Tanzania with the other three countries were below 0.10, data from all four countries were combined for subsequent analyses (Ferguson 2016; Mutuyimana and Maercker 2025).

Means, standard deviations and bivariate correlations among study variables are shown in Tables 2 and 3. Most correlations were significant, although self‐mastery was not associated with reward for application or religiosity.

**TABLE 2:** Means, standard deviations, skewness, kurtosis and Pearson correlation matrix for study variables.

Variable	1	2	3	4	5	6	7	8	9
1. SocCyn	—								
2. RewApp	0.21***	—							
3. SocCom	0.19***	0.58***	—						
4. FatCon	0.25***	0.11***	0.15***	—					
5. Religi	0.19***	0.51***	0.47***	0.17***	—				
6. GSE	−0.15***	0.28***	0.30***	−0.16***	0.23***	—			
7. Master	−0.14***	0.02	0.08**	−0.10***	0.02	0.34***	—		
8. PTSD	0.17***	−0.09**	−0.10***	0.25***	−0.12***	−0.41***	−0.23***	—	
9. DSO	0.09**	−0.16***	−0.24***	0.07*	−0.23***	−0.41***	−0.34***	0.47***	—
10. CPTSD	0.15***	−0.15***	−0.20***	0.18***	−0.20***	−0.48***	−0.33***	0.85***	0.87***
11. Cognit	0.14***	−0.17***	−0.18***	0.13***	−0.18***	−0.39***	−0.17***	0.35***	0.37***
12. FraMin	0.15***	−0.15***	−0.16***	0.29***	−0.10***	−0.31***	−0.12***	0.39***	0.24***
13. BodRel	0.14***	−0.13***	−0.23***	0.08**	−0.19***	−0.40***	−0.19***	0.42***	0.39***
14. Growth	0.08**	0.40***	0.40***	0.05	0.35***	0.29***	0.10***	−0.09**	−0.25***
15. FamStr	0.17***	−0.15***	−0.18***	0.14***	−0.19***	−0.47***	−0.22***	0.38***	0.38***
Mean	3.26	11.09	11.21	6.04	10.75	29.98	15.30	9.27	8.75
SD	1.09	2.67	2.75	1.99	2.88	6.13	3.48	5.56	6.04
Sk	−0.15	−0.47	−0.58	0.01	−0.36	0.21	0.23	−0.06	0.22
Ku	−0.61	−0.01	−0.11	−0.50	−0.34	−0.30	0.14	−0.64	−0.95

Abbreviations: BodRel = body‐related symptoms, Cognit = cognitive symptoms, CPTSD = complex posttraumatic stress disorder, DSO = disturbances in self‐organization, FamStr = family stress, FatCon = fate control, FraMin = frame of mind, GSE = general self‐efficacy, Ku = kurtosis, Master = self‐mastery, PTSD = posttraumatic stress disorder, Religi = religiosity, RewApp = reward for application, Sk = skewness, SocCom = social complexity, SocCyn = social cynicism.

* *p* < 0.05.

** *p* < 0.01.

*** *p* < 0.001.

**TABLE 3:** Means, standard deviations, skewness, kurtosis and Pearson correlation matrix for study variables 10–15.

Variable	10	11	12	13	14	15
10. CPTSD	—					
11. Cognit	0.42***	—				
12. FraMin	0.37***	0.45***	—			
13. BodRel	0.47***	0.56***	0.38***	—		
14. Growth	−0.20***	−0.30***	−0.16***	−0.25***	—	
15. FamStr	0.44***	0.56***	0.43***	0.58***	−0.29***	—
Mean	18.02	4.45	4.97	4.78	8.24	4.46
SD	9.97	3.03	2.91	3.12	2.90	2.85
Sk	0.03	0.32	0.22	0.16	−0.74	0.31
Ku	−0.67	−0.62	−0.62	−0.81	0.09	−0.53

*Note:* See Table 2 for full note and abbreviations.

### 3.2 Hierarchical Multiple Regressions and LMG Analysis

To assess whether SAS subfactors explained additional variance in CSTs, PTSD and DSO beyond intrapersonal variables, we conducted hierarchical multiple regressions and calculated LMG for five CST subfactors, PTSD and DSO. In each regression, intrapersonal predictors (GSE and self‐mastery) were entered in Step 1, followed by SAS subfactors (social cynicism, reward for application, social complexity, fate control and religiosity) in Step 2, allowing evaluation of the incremental explanatory power of social axioms.

Across all seven dependent variables, adding SAS subfactors significantly increased explained variance (Δ*R* ^2^), demonstrating that social axioms contributed additional predictive value beyond GSE and self‐mastery. For instance, in predicting family stress, the Step 1 model with only intrapersonal predictors accounted for 22% of the variance, *F*(2, 1205) = 174.40, *p <* 0.001. Inclusion of SAS variables in Step 2 produced a significant Δ*R* ^2^ of 0.026, *F*(5, 1200) = 8.05, *p <* 0.001. Similar improvements were observed for the remaining six outcomes (see Table 4).

**TABLE 4:** Summary of hierarchical regression and LMG relative importance analyses predicting CSTs variables.

Dependent variable	*R* ^2^ step 1 (intra)	Δ*R* ^2^ step 2 (SAS)	*F* change (Δ*R* ^2^ test)	*p*	Sum of LMGs (SAS)	Sum of LMGs (SAS) mean (/5)	Sum of LMGs (intra)	Sum of LMGs (intra) mean (/2)
Cognit	0.15	0.03	8.48	< 0.001***	0.38	0.08	0.62	0.31
FraMin	0.10	0.08	24.75	< 0.001***	0.66	0.13	0.34	0.17
BodRel	0.16	0.03	10.03	< 0.001***	0.37	0.07	0.63	0.32
Growth	0.09	0.16	49.12	< 0.001***	0.81	0.16	0.19	0.10
FamStr	0.22	0.03	8.05	< 0.001***	0.29	0.06	0.71	0.36
PTSD	0.18	0.04	13.70	< 0.001***	0.36	0.07	0.64	0.32
DSO	0.21	0.03	10.18	< 0.001***	0.29	0.06	0.71	0.36

*Note:* Δ*R* ^2^ values indicate the change in explained variance after entering social axioms in Step 2.

Abbreviations: BodRel = body‐related symptoms, Cognit = cognitive symptoms, CSTs = cultural scripts of trauma, DSO = disturbances in self‐organization, FamStr = family stress, FraMin = frame of mind, Intra = intrapersonal variables (including general self‐efficacy and self‐mastery), LMG = Lindeman, Merenda and Gold method (used for relative importance analysis), PTSD = posttraumatic stress disorder, SAS = Social Axioms Survey.

* *p <* 0.05.

** *p <* 0.01.

*** *p <* 0.001.

To further evaluate the contribution of individual predictors, LMG relative importance analyses were conducted for all seven outcomes. SAS accounted for a larger share of explained variance in growth compared with intrapersonal variables (see Table 4). Further examination of growth revealed that the SAS subscales social complexity, reward for application and religiosity contributed most to its variance, explaining 30%, 30% and 20% of the variance, respectively.

### 3.3 Structural Equation Models

As part of the SEM analysis, we examined whether the data satisfied the normality and multicollinearity assumption (Kline 2016). Skewness and kurtosis values for study variables are reported in Table 5, with ±3 as the cutoff for skewness (Chou and Bentler 1995), indicating that the normality assumption was met. Multicollinearity was assessed via variance inflation factor (VIF), with all values below 5, suggesting that it was not problematic.

**TABLE 5:** Fit indices for two structural equation models.

Model	χ^2^	df	CFI	TLI	RMSEA (90% CI)	SRMR	AIC	BIC
**Initial model (final model)**	**3096.73**	**1235**	**0.92**	**0.91**	**0.035 (0.034, 0.037)**	**0.050**	**183564.91**	**184563.86**
Competitive model	3767.26	1271	0.89	0.87	0.040 (0.039, 0.042)	0.053	184163.44	184978.91

*Note:* All χ^2^ results are statistically significant (*p* < 0.001); best fitting models in bold.

An initial SEM model was constructed (Figure 1) and demonstrated good fit (Table 5). A competitive model was also tested, creating CSTI‐Negative to summarize negative CSTI aspects (cognitive symptoms, frame of mind, body‐related symptoms and family stress) versus CSTI‐Growth. This competitive model showed only borderline acceptable fit (Table 5). The initial model, with higher CFI and TLI and lower RMSEA, SRMR, AIC and BIC values, was therefore selected as the final model.

**FIGURE 2:**
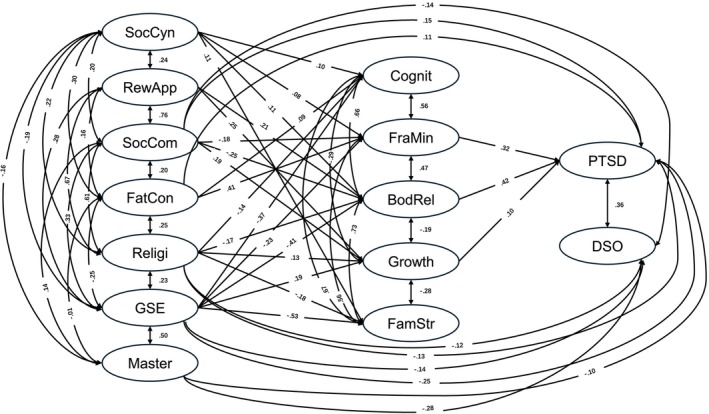
Final structural equation modelling diagram. BodRel = body‐related symptoms, Cognit = cognitive symptoms, DSO = disturbances in self‐organization, FamStr = family stress, FatCon = fate control, FraMin = frame of mind, GSE = general self‐efficacy, Master = self‐mastery, PTSD = posttraumatic stress disorder, Religi = religiosity, RewApp = reward for application, SocCom = social complexity, SocCyn = social cynicism.

Analysis of the final model (Figure 2) confirmed the latent structure, with all factor loadings positive, significant (*p <* 0.001) and robust (> 0.50). The model explained 47% of the variance in PTSD, 41% in DSO, 27% in cognitive symptoms, 34% in frame of mind, 34% in body‐related symptoms, 36% in growth and 44% in family stress. Significant standardized paths between social axioms, intrapersonal variables and CSTs are shown in Figure 2.

Bootstrapping analyses indicated significant mediating effects of social axioms on PTSD via CSTs. Specifically, the indirect effects were: social cynicism → body‐related symptoms → PTSD (*β* = 0.05, *p <* 0.05), social complexity → body‐related symptoms → PTSD (*β* = −0.11, *p <* 0.05), fate control → frame of mind → PTSD (*β* = 0.13, *p <* 0.01) and religiosity → body‐related symptoms → PTSD (*β* = −0.07, *p <* 0.05). For intrapersonal variables, GSE exerted significant indirect effects on PTSD through frame of mind (*β* = −0.08, *p <* 0.05) and body‐related symptoms (*β* = −0.17, *p <* 0.01).

Direct effects were also observed: religiosity on PTSD and DSO (*β* = −0.13 and *β* = −0.12, both *p <* 0.05) and GSE on PTSD and DSO (*β* = −0.25, *p <* 0.01; *β* = −0.14, *p <* 0.05). Frame of mind and body‐related symptoms were positively associated with PTSD (*β* = 0.32 and *β* = 0.42, both *p <* 0.001). Further details are provided in Table A2 (Appendix).

## 4 Discussion

Investigating CST is still a new approach in East Africa and beyond (Chentsova‐Dutton and Maercker 2019). The current study extends the work of Mutuyimana and Maercker (2026), who developed and validated the CSTI in East Africa, by employing this measure to investigate how its domains relate to social axioms, intrapersonal variables, as well as PTSD and CPTSD symptom severity. Deriving from the opinions that culture acts as an important factor that shapes people's thoughts, perceptions, feelings and behaviours (Agbayani‐Siewert et al. 1999; Napier et al. 2014; Zhou et al. 2016), this study examined the relationships among CSTs, social axioms, intrapersonal variables (GSE and self‐mastery), PTSD and DSO among trauma survivors in East Africa. In brief, we found that social axioms (social complexity, reward for application and religiosity) provided additional predictive value for growth beyond GSE and self‐mastery. Moreover, the CST clusters ‘frame‐of‐mind symptoms’ and ‘body‐related symptoms’ were positively associated with PTSD severity. Finally, social axioms indirectly contributed to PTSD and CPTSD symptoms through their influence on CSTs.

### 4.1 Social Axioms Predicted Posttraumatic Growth (PTG) in East Africa

In this study, the construct labelled ‘growth’ was derived from the CSTI and reflects culturally specific forms of positive change identified among East African trauma survivors. This construct is related to—but not conceptually identical to—the broader notion of PTG described in the literature. To situate our findings within existing theoretical frameworks, we draw on PTG research to interpret the meaning and potential mechanisms of growth. In doing so, we refer to explanations such as ‘illusory’ or ‘struggle‐based’ PTG (Maercker and Zoellner 2004; Zoellner and Maercker 2006) to help contextualize how culturally grounded growth may emerge and how it aligns with or diverges from established PTG models. The first hypothesis was only partially supported. Social axioms more accurately predicted growth signs than GSE and self‐mastery. Among the social axioms, social complexity, reward for application and religiosity emerged as the strongest predictors. These findings align with previous research showing positive associations between these social axioms and posttraumatic growth (Nalipay et al. 2016; Nalipay et al. 2017). Extending previous findings, the current study demonstrates that these social axioms are also important for culturally grounded indicators of PTG among East African trauma survivors. Furthermore, these findings underscore the role of distal culture. As distal cultural elements, social axioms appear to support deliberate meaning‐making processes that facilitate posttraumatic growth. Broader cultural belief systems play a more central role in posttraumatic growth than individual‐level belief systems.

### 4.2 CSTs Predicted PTSD in Culturally Specific Ways

The second hypothesis was partly supported. Structural equation modelling revealed positive associations between frame‐of‐mind symptoms, body‐related symptoms and PTSD severity. Frame‐of‐mind symptoms are characterized by lacking future planning due to persistent uncertainties and doubts about the reliability of support from others (Mutuyimana and Maercker 2026). Individuals with more severe PTSD symptoms often experience impaired ability to envision or plan for the future (American Psychiatric Association 2022). Reduced confidence in long‐term stability and social support networks may reflect PTSD features such as mistrust and social withdrawal. Perceived social support is both a protective factor against PTSD development and an important component of posttrauma recovery (Ozer et al. 2003). Individuals who lack confidence in social support systems may find it harder to regulate distress and engage in adaptive coping, thereby intensifying PTSD severity. Somatic symptoms are commonly observed in individuals with posttraumatic stress (e.g., Ao and Graham 2021; Kratzer et al. 2022). The positive relationship between body‐related symptoms and PTSD severity likely reflects the somatic nature of trauma responses among East African survivors. PTSD is often associated with heightened bodily arousal, physical tension and somatization.

### 4.3 CSTs Mediated the Impact of Cultural Values on PTSD

The role of social axioms as indirect contributors to PTSD and CPTSD symptoms via CSTs was examined exploratorily. These findings revealed divergent cultural syndrome pathways to PTSD. Indirect effect analyses indicated that the relationships between social axioms (including social cynicism, social complexity, fate control and religiosity) and PTSD severity were partially mediated by frame of mind and body‐related symptoms. Notably, social cynicism was associated with increased PTSD severity through heightened body‐related symptoms. Since cynicism is negatively related to self‐control (Jackson et al. 2020) and individuals with higher self‐control tend to have better physical health (Valkenburg 2018), this may extend to body‐related trauma sequelae.

Social complexity, by contrast, may foster greater cognitive flexibility. Individuals with higher psychological flexibility tend to report fewer somatic symptoms (Jackson et al. 2020), which may explain the association with lower PTSD severity. Religiosity was negatively associated with PTSD symptoms and mediated through reduced body‐related symptoms. This is consistent with research highlighting the resilience‐enhancing role of faith‐based belief systems (Green and Meadows 2024; Isiwele et al. 2025). Conversely, fate control was positively associated with frame‐of‐mind disruptions, indirectly associated with increased PTSD symptom severity. This pattern suggests a culturally reinforced sense of helplessness (Bond, Leung, Au, Tong, and Chemonges‐Nielson 2004; Leung et al. 2002). These findings underscore the important role of culture in shaping and manifesting posttraumatic symptoms (Heim et al. 2022; Van Rooyen and Nqweni 2012).

The structural equation model demonstrated good fit and provides a theoretically grounded framework for how broader belief systems and individual agency shape CSTs, which in turn predict PTSD severity. First‐layer factors such as social complexity, fate control and GSE exerted both direct and indirect effects on PTSD, mainly through CSTs including frame of mind, body‐related symptoms and growth. These results suggest that higher self‐efficacy may buffer trauma impact through adaptive processing, whereas fatalistic beliefs may undermine coping resources and increase PTSD via maladaptive cognitive or somatic responses. At the second layer, culturally specific responses emerged: disruptions in worldview, relationships and bodily experiences were linked to more severe PTSD symptoms. Notably, growth also correlated positively with PTSD, supporting the idea that growth and distress can coexist, consistent with prior work on ‘illusory’ or ‘struggle‐based’ PTG (Maercker and Zoellner 2004; Zoellner and Maercker 2006). Overall, the model illustrates a layered process in which foundational beliefs and personal agency shape intermediate somatic and cognitive outcomes that culminate in PTSD severity. This aligns with multilevel theoretical perspectives such as the social‐cognitive processing model (Lepore 2001) and underscores that trauma sequelae reflect not only exposure but also interpretation and regulation of internal and social experiences. Importantly, our findings support the view that trauma responses vary across cultural contexts and may include sequelae beyond established PTSD criteria (Maercker and Hecker 2016; Maercker and Horn 2013; Patel and Hall 2021). Trauma sequelae may include both pan‐cultural as well as culture‐specific phenomena (Chentsova‐Dutton and Maercker 2019; Heim et al. 2022). CSTs encompass posttraumatic sequalae, which are not necessarily reflected in existing PTSD criteria but that nonetheless require clinical attention (Mutuyimana and Maercker 2023). In the current model, all CSTs were associated with each other, supporting previous findings that all mental categories of trauma sequelae in East Africa cluster closely together (Mutuyimana and Maercker 2023). This further reflects the concept of cultural scripts of traumatic stress, which suggests that CST sequelae include symptoms that are widely believed to be associated with each other (Chentsova‐Dutton and Maercker 2019).

The findings align with prior CSTs research across cultural settings. For instance, trauma survivors in Switzerland emphasized body‐related phenomena and growth (Bachem et al. 2024), whereas East African participants highlighted family stress, reflecting communal cultural values (Mutuyimana and Maercker 2023). Such variations underscore that cultural concepts of distress may capture the posttraumatic reality of non‐Western survivors more accurately than Western diagnostic criteria for PTSD and CPTSD (Hinton et al. 2019). In our SEM analysis, cognitive symptoms and family stress emerged as distinct from PTSD and DSO severity, suggesting culturally specific sequelae in East Africa that are not encompassed by ICD‐11. These findings extend and reinforce previous CSTs research (Mutuyimana and Maercker 2023, 2026).

In summary, this study demonstrates that cultural values contribute to PTSD severity both directly and indirectly through their influence on CSTs. The cultural scripts of traumatic stress framework offers a valuable lens for understanding how cultural backgrounds shape posttraumatic sequelae. By highlighting CSTs specific to the East African context, our findings show that culturally relevant responses extend beyond the symptom profiles described in DSM‐5‐TR and ICD‐11. The validated structural model provides an integrative SEM‐based account of how cultural values and intrapersonal factors interact to predict PTSD, thereby advancing quantitative evidence for linking CSTs with PTSD severity. These results support theoretical perspectives that culture‐specific factors shape the perception and manifestation of stress, giving rise to distinct syndromes (Eberle et al. 2024). Consistent with prior research (Kessler et al. 2017; Maercker and Heim 2019), the findings underscore the substantial role of culture in trauma responses and highlight the need for further empirical investigation into CSTs and related cultural syndromes.

### 4.4 Limitations and Future Research

Several limitations should be noted. First, the cross‐sectional design precludes conclusions about causality or temporal sequencing between cultural values, intrapersonal factors, CSTs and PTSD severity—an important element of CSTs theory (Chentsova‐Dutton and Maercker 2019). Second, reliance on self‐report rather than clinical interviews may limit inferences beyond participants' perceptions and underestimate cultural aspects of trauma. Third, although the sample was regionally representative of East African adults, the use of online survey platforms may have led to an overrepresentation of more educated or well‐resourced individuals. Fourth, this study only investigated common and universally recognized traumatic events; these events are not cultural‐specific (be perceived as traumatic or stressful only within specific cultural frameworks).

Future research should employ longitudinal or experimental designs to clarify causal pathways among cultural values, CSTs, and PTSD/DSO severity. Broader cultural variables should also be examined to identify additional factors influencing CSTs. Moreover, cross‐cultural investigations and validation of CSTs are needed to develop culturally specific models of trauma sequelae, which would enhance the refinement of diagnostic frameworks and inform culturally sensitive interventions. Finally, certain events may be perceived as traumatic or stressful only within specific cultural frameworks. This is an important area for further investigation, and future studies could examine how culturally specific appraisals shape the definition and experience of traumatic events.

### 4.5 Theoretical and Practical Contributions

Despite these limitations, the current study holds significant implications for both theoretical and practical realms of trauma and culture. The empirical findings supported the validity of the CSTs in East Africa not only in relation to PTSD severity but also in relation to cultural values. The study revealed distinct relationships between different CSTs and social axioms, supporting the notion that cultural values act as significant risk and protective factors following traumatic events (Maercker and Hecker 2016; Maercker and Horn 2013). The findings also supported the notion that CSTs share common characteristics and are interrelated (Chentsova‐Dutton and Maercker 2019; Mutuyimana and Maercker 2023). In turn, this aligns with previous findings indicating positive correlations among various psychopathological symptoms (Eberle and Maercker 2023; Forbes et al. 2015; Haselgruber et al. 2020). CSTI is a culturally informed, complementary tool to established universal measures such as the ITQ. It is designed to capture culturally specific trauma sequelae that may not be fully reflected in universal diagnostic instruments.

The study results support the notion that CSTs in East Africa are important trauma sequelae, which go beyond PTSD and DSO of complex PTSD symptoms. Thus, from a practical perspective, clinicians should hold the knowledge of the association between cultural values, which may influence trauma sequelae and the cultural‐specific traumatic responses (such as CSTs) that are not included in the description of PTSD and DSO symptoms. The CSTI can provide clinicians with a more nuanced understanding of trauma responses across cultural contexts. Additionally, clinicians should take the individuals' cultural beliefs within their specific cultural context into account when delivering trauma‐informed care. However, evaluation of the long‐term outcomes of these assessments and interventions is needed, as the cultural‐specific trauma sequelae may also be shaped by additional contributing factors, such as sociopolitical conditions (e.g., marginalization or discrimination) and historical contexts (e.g., colonial histories or genocide).

## 5 Conclusion

This study quantitatively examined the interplay of cultural, intrapersonal and trauma‐related factors among East African trauma survivors through the lens of CSTs, extending the work of Mutuyimana and Maercker (2026). We investigated the predictive role of social axioms, associations between CSTs and PTSD/DSO symptoms and the mediating role of CSTs in linking cultural values to trauma outcomes.

Findings indicate that social axioms—particularly complexity, reward for application and religiosity—were stronger predictors of growth‐related CSTs than individual factors such as GSE and self‐mastery, highlighting the influence of broader cultural belief systems on posttraumatic adaptation. CSTs such as frame of mind and body‐related symptoms were positively associated with PTSD severity, suggesting culturally rooted expressions of trauma not fully captured by standard diagnostic criteria. Mediation analyses further demonstrated that CSTs serve as pathways through which cultural values affect PTSD symptoms, reflecting both protective and risk processes.

Overall, these results support a layered, culturally informed model of trauma adaptation in which pan‐cultural and culturally specific sequelae interact with foundational belief systems. Structural equation modelling validated this framework in the East African context, underscoring the need for culturally sensitive diagnostic and clinical approaches. Future research should employ longitudinal designs to examine the temporal and causal dynamics of CSTs and extend cross‐cultural validation to develop inclusive trauma models. This study advances CST theory by empirically linking cultural belief systems to trauma‐related outcomes and emphasizes the importance of culturally grounded assessments and interventions in posttraumatic mental health care.

## Author Contributions

Conceptualization: Andreas Maercker, Celestin Mutuyimana. Data curation: Celestin Mutuyimana. Data analysis: Yucong Wen. Visualization: Yucong Wen. Supervision: Andreas Maercker. Writing – original draft: Yucong Wen. Writing – review and editing: Andreas Maercker, Nathanael Adank.

## Funding

This work was supported by University of Zurich during the process of data collection. No external or formal grant funding was received.

## Ethics Statement

This study was approved by the Ethics Committee of the University of Rwanda, College of Medicine and Health Sciences, Approval No. 396/CMHS IRB/2022, and the Ethics Committee of Daystar University Kenya, Approval No. DU‐ISERC/09/05/2023/00086.

## Consent

Participants provided electronic informed consent forms to participate in this study.

## Data Availability

The data that support the findings of this study are available from the corresponding author upon reasonable request.

## Appendix A

**TABLE A1:** Difference of symptoms between four countries.

DV	Country	*F*(3, 1204)	*p*	M ± SD	Significant pairwise differences (Tamhane's T2)
PTSD	Rwanda	49.956	< 0.001	6.77 ± 5.97	Rwanda < Kenya (*p <* 0.001), Rwanda < Tanzania (*p <* 0.001), Rwanda < Uganda (*p <* 0.01)
	Kenya			10.10 ± 6.15	Kenya > Rwanda (*p <* 0.001), Kenya < Tanzania (*p <* 0.05), Kenya > Uganda (*p <* 0.01)
	Tanzania			11.34 ± 3.82	Tanzania > Rwanda (*p <* 0.001), Tanzania > Kenya (*p <* 0.05), Tanzania > Uganda (*p <* 0.001)
	Uganda			8.36 ± 5.45	Uganda > Rwanda (*p <* 0.01), Uganda < Kenya (*p <* 0.01), Uganda < Tanzania (*p <* 0.001)
RE	Rwanda	30.939	< 0.001	1.92 ± 2.13	Rwanda < Kenya (*p <* 0.001), Rwanda < Tanzania (*p <* 0.001), Rwanda < Uganda (*p <* 0.01)
	Kenya			2.85 ± 2.36	Kenya > Rwanda (*p <* 0.001), Kenya < Tanzania (*p <* 0.05)
	Tanzania			3.37 ± 1.79	Tanzania > Rwanda (*p <* 0.001), Tanzania > Kenya (*p <* 0.05), Tanzania > Uganda (*p <* 0.001)
	Uganda			2.51 ± 2.07	Uganda > Rwanda (*p <* 0.01), Uganda < Tanzania (*p <* 0.001)
AV	Rwanda	20.649	< 0.001	2.56 ± 2.40	Rwanda < Kenya (*p <* 0.001), Rwanda < Tanzania (*p <* 0.001)
	Kenya			3.70 ± 2.43	Kenya > Rwanda (*p <* 0.001), Kenya > Uganda (*p <* 0.01)
	Tanzania			3.72 ± 1.88	Tanzania > Rwanda (*p <* 0.001), Tanzania > Uganda (*p <* 0.001)
	Uganda			3.00 ± 2.28	Uganda < Kenya (*p <* 0.01), Uganda < Tanzania (*p <* 0.001)
ST	Rwanda	56.309	< 0.001	2.29 ± 2.38	Rwanda < Kenya (*p <* 0.001), Rwanda < Tanzania (*p <* 0.001), Rwanda < Uganda (*p <* 0.05)
	Kenya			3.56 ± 2.56	Kenya > Rwanda (*p <* 0.001), Kenya < Tanzania (*p <* 0.01), Kenya > Uganda (*p <* 0.01)
	Tanzania			4.25 ± 1.71	Tanzania > Rwanda (*p <* 0.001), Tanzania > Kenya (*p <* 0.01), Tanzania > Uganda (*p <* 0.001)
	Uganda			2.84 ± 2.01	Uganda > Rwanda (*p <* 0.05), Uganda < Kenya (*p <* 0.01), Uganda < Tanzania (*p <* 0.001)
DSO	Rwanda	47.723	< 0.001	6.55 ± 5.54	Rwanda < Kenya (*p <* 0.01), Rwanda < Tanzania (*p <* 0.001)
	Kenya			8.32 ± 6.24	Kenya > Rwanda (*p <* 0.01), Kenya < Tanzania (*p <* 0.001)
	Tanzania			11.35 ± 5.43	Tanzania > Rwanda (*p <* 0.001), Tanzania > Kenya (*p <* 0.001), Tanzania > Uganda (*p <* 0.001)
	Uganda			7.67 ± 5.90	Uganda < Tanzania (*p <* 0.001)
AD	Rwanda	41.580	< 0.001	2.36 ± 2.21	Rwanda < Kenya (*p <* 0.001), Rwanda < Tanzania (*p <* 0.001)
	Kenya			3.40 ± 2.30	Kenya > Rwanda (*p <* 0.001), Kenya < Tanzania (*p <* 0.01), Kenya > Uganda (*p <* 0.01)
	Tanzania			3.99 ± 1.87	Tanzania > Rwanda (*p <* 0.001), Tanzania > Kenya (*p <* 0.01), Tanzania > Uganda (*p <* 0.001)
	Uganda			2.74 ± 2.11	Uganda < Kenya (*p <* 0.01), Uganda < Tanzania (*p <* 0.001)
NSC	Rwanda	43.316	< 0.001	2.39 ± 2.17	Rwanda < Tanzania (*p <* 0.001)
	Kenya			2.07 ± 2.53	Kenya < Tanzania (*p <* 0.001)
	Tanzania			3.87 ± 2.29	Tanzania > Rwanda (*p <* 0.001), Tanzania > Kenya (*p <* 0.001), Tanzania > Uganda (*p <* 0.001)
	Uganda			2.39 ± 2.26	Uganda < Tanzania (*p <* 0.001)
DR	Rwanda	35.799	< 0.001	1.80 ± 2.04	Rwanda < Kenya (*p <* 0.001), Rwanda < Tanzania (*p <* 0.001), Rwanda < Uganda (*p <* 0.001)
	Kenya			2.85 ± 2.52	Kenya > Rwanda (*p <* 0.001), Kenya < Tanzania (*p <* 0.01)
	Tanzania			3.49 ± 2.14	Tanzania > Rwanda (*p <* 0.001), Tanzania > Kenya (*p <* 0.01), Tanzania > Uganda (*p <* 0.001)
	Uganda			2.54 ± 2.25	Uganda > Rwanda (*p <* 0.001), Uganda < Tanzania (*p <* 0.001)

Abbreviations: AD= affective dysregulation, AV = avoidance, DR = disturbances in relationships, DSO = disturbance in self‐organization, DV = dependent variables, NSC = negative self‐concept, RE = re‐experiencing, ST = sense of current threat.

**TABLE A2:** Direct and indirect standardized path coefficients derived from final model.

IV	Mediator	DV	Effect type	β	SE	*p*
SocCyn	Cognit	PTSD	Indirect	−0.01	0.01	0.332
SocCyn	Cognit	DSO	Indirect	0.01	0.01	0.167
SocCyn	FraMin	PTSD	Indirect	0.03	0.01	0.082
SocCyn	FraMin	DSO	Indirect	0.00	0.00	0.998
SocCyn	BodRel	PTSD	Indirect	0.05	0.02	0.035*
SocCyn	BodRel	DSO	Indirect	0.02	0.01	0.188
SocCyn	Growth	PTSD	Indirect	−0.00	0.00	0.854
SocCyn	Growth	DSO	Indirect	0.00	0.00	0.893
SocCyn	FamStr	PTSD	Indirect	−0.02	0.01	0.358
SocCyn	FamStr	DSO	Indirect	0.00	0.01	0.801
SocCyn	—	PTSD	Direct	0.02	0.02	0.630
SocCyn	—	DSO	Direct	0.01	0.02	0.867
SocCyn	—	PTSD	Total	0.06	0.02	0.062
SocCyn	—	DSO	Total	0.04	0.02	0.251
RewApp	Cognit	PTSD	Indirect	0.00	0.01	0.708
RewApp	Cognit	DSO	Indirect	−0.01	0.01	0.651
RewApp	FraMin	PTSD	Indirect	−0.02	0.03	0.564
RewApp	FraMin	DSO	Indirect	0.00	0.01	0.999
RewApp	BodRel	PTSD	Indirect	0.09	0.05	0.058
RewApp	BodRel	DSO	Indirect	0.03	0.02	0.210
RewApp	Growth	PTSD	Indirect	0.02	0.02	0.129
RewApp	Growth	DSO	Indirect	−0.01	0.01	0.448
RewApp	FamStr	PTSD	Indirect	−0.01	0.02	0.735
RewApp	FamStr	DSO	Indirect	0.00	0.01	0.898
RewApp	—	PTSD	Direct	−0.05	0.08	0.584
RewApp	—	DSO	Direct	0.11	0.07	0.142
RewApp	—	PTSD	Total	0.05	0.08	0.559
RewApp	—	DSO	Total	0.13	0.06	0.083
SocCom	Cognit	PTSD	Indirect	0.00	0.01	0.758
SocCom	Cognit	DSO	Indirect	−0.00	0.01	0.710
SocCom	FraMin	PTSD	Indirect	−0.06	0.03	0.076
SocCom	FraMin	DSO	Indirect	0.00	0.01	0.998
SocCom	BodRel	PTSD	Indirect	−0.11	0.04	0.040*
SocCom	BodRel	DSO	Indirect	−0.04	0.02	0.200
SocCom	Growth	PTSD	Indirect	0.02	0.01	0.165
SocCom	Growth	DSO	Indirect	−0.01	0.01	0.412
SocCom	FamStr	PTSD	Indirect	0.01	0.02	0.766
SocCom	FamStr	DSO	Indirect	−0.00	0.01	0.914
SocCom	—	PTSD	Direct	0.15	0.08	0.102
SocCom	—	DSO	Direct	−0.14	0.06	0.083
SocCom	—	PTSD	Total	0.01	0.06	0.885
SocCom	—	DSO	Total	−0.19	0.05	0.007**
FatCon	Cognit	PTSD	Indirect	−0.01	0.01	0.437
FatCon	Cognit	DSO	Indirect	0.01	0.01	0.289
FatCon	FraMin	PTSD	Indirect	0.13	0.04	0.001**
FatCon	FraMin	DSO	Indirect	0.00	0.03	0.998
FatCon	BodRel	PTSD	Indirect	0.01	0.02	0.641
FatCon	BodRel	DSO	Indirect	0.00	0.01	0.671
FatCon	Growth	PTSD	Indirect	0.00	0.01	0.693
FatCon	Growth	DSO	Indirect	−0.00	0.00	0.789
FatCon	FamStr	PTSD	Indirect	−0.01	0.01	0.523
FatCon	FamStr	DSO	Indirect	0.00	0.01	0.851
FatCon	—	PTSD	Direct	0.11	0.06	0.089
FatCon	—	DSO	Direct	0.00	0.04	0.975
FatCon	—	PTSD	Total	0.24	0.05	< 0.001***
FatCon	—	DSO	Total	0.018	0.03	0.689
Religi	Cognit	PTSD	Indirect	0.013	0.01	0.370
Religi	Cognit	DSO	Indirect	−0.02	0.01	0.200
Religi	FraMin	PTSD	Indirect	−0.02	0.02	0.468
Religi	FraMin	DSO	Indirect	0.00	0.01	0.999
Religi	BodRel	PTSD	Indirect	−0.07	0.03	0.025*
Religi	BodRel	DSO	Indirect	−0.03	0.01	0.200
Religi	Growth	PTSD	Indirect	0.01	0.01	0.156
Religi	Growth	DSO	Indirect	−0.01	0.01	0.437
Religi	FamStr	PTSD	Indirect	0.03	0.03	0.384
Religi	FamStr	DSO	Indirect	−0.01	0.02	0.814
Religi	—	PTSD	Direct	−0.13	0.05	0.028*
Religi	—	DSO	Direct	−0.12	0.04	0.021*
Religi	—	PTSD	Total	−0.17	0.05	0.003**
Religi	—	DSO	Total	−0.18	0.04	0.001**
GSE	Cognit	PTSD	Indirect	0.03	0.10	0.336
GSE	Cognit	DSO	Indirect	−0.05	0.08	0.143
GSE	FraMin	PTSD	Indirect	−0.08	0.10	0.022*
GSE	FraMin	DSO	Indirect	0.00	0.05	0.998
GSE	BodRel	PTSD	Indirect	−0.17	0.18	0.006**
GSE	BodRel	DSO	Indirect	−0.06	0.11	0.161
GSE	Growth	PTSD	Indirect	0.02	0.04	0.122
GSE	Growth	DSO	Indirect	−0.01	0.03	0.438
GSE	FamStr	PTSD	Indirect	0.09	0.27	0.352
GSE	FamStr	DSO	Indirect	−0.02	0.18	0.804
GSE	—	PTSD	Direct	−0.25	0.25	0.003**
GSE	—	DSO	Direct	−0.14	0.16	0.034*
GSE	—	PTSD	Total	−0.36	0.24	< 0.001***
GSE	—	DSO	Total	−0.27	0.16	< 0.001***
Master	Cognit	PTSD	Indirect	0.00	0.01	0.667
Master	Cognit	DSO	Indirect	−0.00	0.01	0.621
Master	FraMin	PTSD	Indirect	0.02	0.02	0.422
Master	FraMin	DSO	Indirect	0.00	0.01	0.999
Master	BodRel	PTSD	Indirect	−0.02	0.03	0.416
Master	BodRel	DSO	Indirect	−0.01	0.01	0.469
Master	Growth	PTSD	Indirect	0.00	0.01	0.686
Master	Growth	DSO	Indirect	−0.00	0.00	0.769
Master	FamStr	PTSD	Indirect	0.01	0.02	0.587
Master	FamStr	DSO	Indirect	−0.00	0.01	0.861
Master	—	PTSD	Direct	−0.10	0.06	0.042*
Master	—	DSO	Direct	−0.28	0.05	< 0.001***
Master	—	PTSD	Total	−0.10	0.06	0.054
Master	—	DSO	Total	−0.29	0.05	< 0.001***
Cognit	—	PTSD	Direct	−0.09	0.08	0.298
Cognit	—	DSO	Direct	0.12	0.06	0.117
FraMin	—	PTSD	Direct	0.32	0.09	< 0.001***
FraMin	—	DSO	Direct	0.00	0.06	0.998
BodRel	—	PTSD	Direct	0.42	0.10	< 0.001***
BodRel	—	DSO	Direct	0.14	0.07	0.139
Growth	—	PTSD	Direct	0.10	0.06	0.072
Growth	—	DSO	Direct	−0.05	0.05	0.380
FamStr	—	PTSD	Direct	−0.16	0.15	0.314
FamStr	—	DSO	Direct	0.03	0.11	0.798

Abbreviations: BodRel = body‐related symptoms, Cognit = cognitive symptoms, DSO = disturbances in self‐organization, DV = dependent variables, FamStr = family stress, FatCon = fate control, FraMin = frame of mind, GSE = general self‐efficacy, IV = independent variables, Master = self‐mastery, PTSD = posttraumatic stress disorder, Religi = religiosity, RewApp = reward for application, SE = standard error, SocCom = social complexity, SocCyn = social cynicism, *β* = standardized effect.

* *p* < 0.05.

** *p* < 0.01.

*** *p* < 0.001.
